# Atypical Presentation of Myocardial Infarction in a Young Patient With Polycystic Ovarian Syndrome

**DOI:** 10.7759/cureus.9494

**Published:** 2020-07-31

**Authors:** Shobha Mandal, Ravi R Pradhan, Barbara Mols Kowalczewski

**Affiliations:** 1 Internal Medicine, Guthrie Robert Packer Hospital, Sayre, USA; 2 Internal Medicine, Tribhuvan University Institute of Medicine, Kathmandu, NPL; 3 Endocrinology, Guthrie Robert Packer Hospital, Sayre, USA

**Keywords:** polycystic ovarian syndrome, pcos, young female, atypical back pain, myocardial infarction, primary percutaneous coronary intervention

## Abstract

Polycystic ovarian syndrome (PCOS) is one of the common endocrinopathy noted in women of childbearing age groups. Patients with PCOS have increased cardiovascular risk factors compared with age-matched control; hence, these patients are believed to be at an increased risk for cardiovascular events. Here, we report a case of a young female with PCOS, who presented with atypical back pain in the thoracic region. Initially, her electrocardiogram (EKG) and troponin were normal; hence, it was thought to be a muscle spasm but the back pain continued; repeat EKG and troponin came abnormal suggestive of myocardial infarction (MI). The patient underwent primary percutaneous coronary intervention and was discharged on dual antiplatelet therapy. MI is common in patients with PCOS. MI is the most important differential diagnosis in any patients of PCOS presenting with chest pain or back pain. Early diagnosis and prompt treatment of MI in patients with PCOS prevent adverse outcomes.

## Introduction

Polycystic ovarian syndrome (PCOS) is one of the common endocrinopathy noted in women of childbearing age groups, with a prevalence ranging from 4% to 12% globally [1]. Patients with PCOS have insulin resistance, dyslipidemia, obesity, androgen excess, and infertility [2-4]. All these are risk factors for cardiovascular diseases. Because of association with multiple metabolic risk factors in patients with PCOS at a young age, there is an increased chance of developing cardiovascular diseases in this group of patients compared to their age-matched controls [5]. However, evidence generated so far is inconclusive for the risk of MI, and cardiovascular risk profiles may vary with PCOS phenotype, age, ethnicity, and body mass index (BMI). A prospective study conducted by Dahlgren et al. in 1992 showed that the risk of myocardial infarction (MI) was seven times more in peri- and postmenopausal women with PCOS compared to healthy controls [4]. Here, we present a case of a young woman with PCOS presenting with atypical back pain in the thoracic region, which was later diagnosed as MI.

## Case presentation

A 36-year-old, Caucasian female, nonsmoker, with a past medical history of PCOS, anxiety, and depression came to the ED with a complaint of back pain in the thoracic region for 10 days. The pain started suddenly after she lifted a typewriter weighing 60 pounds. The pain was throbbing in nature, 5/10 in intensity, radiating to both arms, worsened with any movement, and lasted for several hours. She was taking over-the-counter tylenol with minimal improvement in the pain. She came to the ED as the pain persisted. On arrival, she rated her pain as severe. She was vitally stable and general physical exam was benign. Routine laboratory workup and troponin were within normal limits. She was suspected to have muscle spasm of the back and was treated with stat doses of ketorolac 30 mg intramuscularly, cyclobenzaprine 10 mg orally as well as a lidocaine transdermal patch. She reported improvement in her back pain and was discharged on cyclobenzaprine 10 mg orally three times a day for seven days. After reaching home she started having the back pain again without any improvement with muscle relaxant (cyclobenzaprine). She also reported having a new onset of nausea and one episode of vomiting. However, she did not have a fever, cough, hemoptysis, and shortness of breath. She came to the ED for further evaluation. On detailed discussion, she reported that her father and grandfather had MI in their fifties, and mother died at the age of 24 during childbirth due to unknown reasons.

On physical examination, the patient appeared anxious. The temperature was 36.7°C (normal = 36.5°C-37.3°C), pulse 121 beats per minute (normal = 60-100 beats per minute), blood pressure 140/93 mmHg ( normal < 120/80 mmHg), respiratory rate 20 breaths per minute (normal = 12-18 breaths per minute), and oxygen saturation 96% (normal = 95%-100%) while she was breathing ambient air. The body mass index (BMI) was 34.6 kg/m² (weight: 96.4 kg, height: 1.67 m). There was mild tenderness in the thoracic spine at the T1-T3 level. The remainder of the physical examination was unchanged. Differential diagnosis included musculoskeletal pain, infectious process (spinal epidural abscess/pericarditis), aortic dissection, pulmonary embolism, and MI.

The details of lab work investigations are shown in Table 1.

**Table 1:** Laboratory parameters of the patient on day 1 and day 2. NA: Not available; BNP: Brain natriuretic peptide; CPK: Creatine phosphokinase; INR: International normalization ratio; TSH: Thyroid stimulating hormone; HDL: High density lipoprotein, LDL: Low density lipoprotein

Parameters	Reference range, adults	Day 1	Day 2
Platelets (per mm³)	182000-369000	358000	351000
White cell count (per mm³)	4000-11000	15710	17240
Neutrophils (%)	34.0-71.1	76.9	78.6
Lymphocytes (%)	19.3-51.7	13.4	12.4
Monocytes (%)	4.7-12.5	8.2	8.1
Eosinophils (%)	0.7-5.8	0.7	0.1
Hemoglobin (g/dL)	11.2-15.7	14.7	12.9
Troponin I (ng/ml)	0.000-0.034	1.43	10.6
N-terminal pro BNP (pg/ml)	<125	1,170	NA
Magnesium (mg/dL)	1.6-2.3	1.9	1.8
Lactic acid (mmol/L)	0.7-2.1	1.7	NA
Random blood sugar (mg/dL)	< 140	121	NA
Creatinine (mg/dL)	0.7-1.2	0.8	0.8
Potassium (mmol/L)	3.5-5.1	4	4.3
Sodium (mmol/L)	134 - 145	140	139
CPK (U/L)	30-135	373	NA
INR	0.8-1.1	0.97	NA
TSH (mIU/L)	0.47-4.68	1.33	NA
C-reactive protein (mg/L)	< 3	0.7	NA
Cholesterol (mg/dL)	< 200	225	NA
Triglycerides (mg/dL)	< 150	145	NA
HDL (mg/dL)	40-59	33	NA
LDL (mg/dL)	100-129	164	NA

Blood culture was negative for any growth. Urine drug screening for amphetamines, barbiturates, benzodiazepines, cannabinoids, cocaine, methadone, opiates, oxycodone, phencyclidine, and propoxyphene was negative.

**Figure 1:**
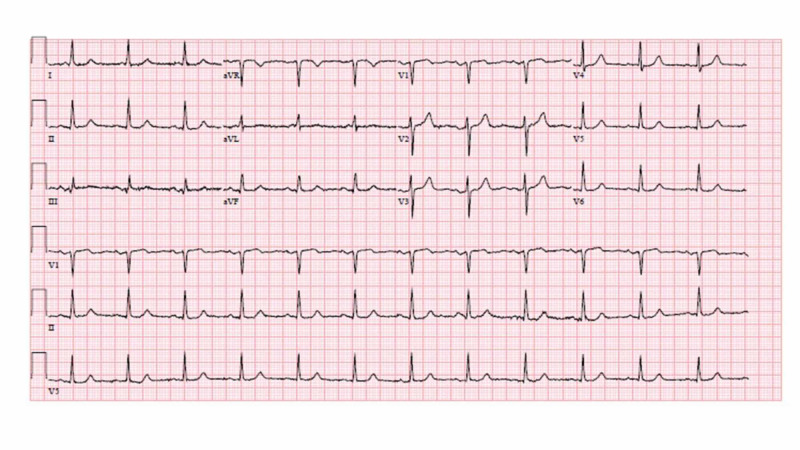
EKG on day 1 with normal findings. EKG, electrocardiogram

CT angiography of the chest, abdomen, and pelvis was negative for pulmonary embolism, aortic aneurysm or dissection, and contrast-enhanced CT scan of the thoracic spine was negative for epidural abscess, fracture, or malalignment. Electrocardiogram (EKG) was initially normal (Figure 1).

**Figure 2:**
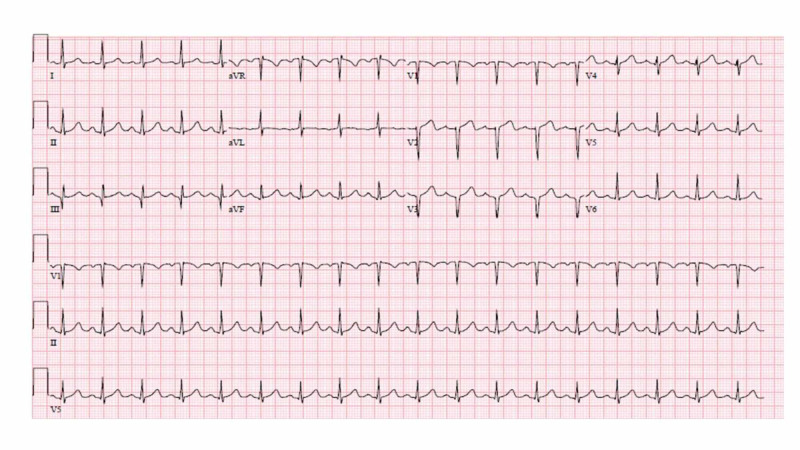
EKG (second day) showing sinus tachycardia, and Q-waves in lead III, and V1 to V3. EKG, electrocardiogram

Repeat EKG the next day showed sinus tachycardia, and Q-wave in lead III, and V1-V3 (Figure 2).

Echocardiogram showed hypokinesis of the left ventricular mid to apical, anterior septum, apical cap, and inferior apical wall.

**Figure 3:**
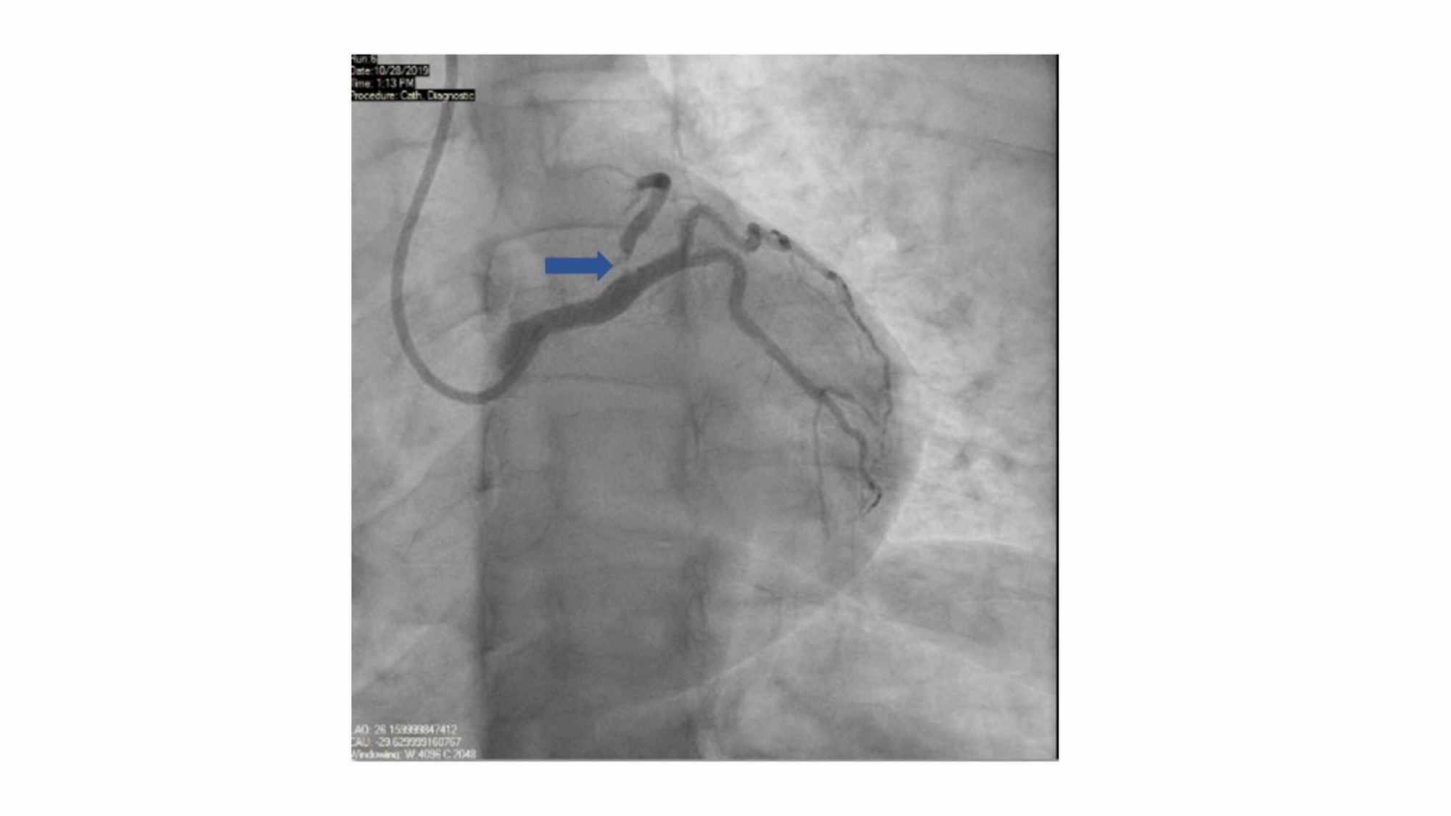
Pre-intervention coronary angiography. Coronary angiography showed 99% ostial LAD artery stenosis, mild luminal irregularities in the left circumflex artery, and RCA dominance. The blue arrow points to the location of stenosis LAD, left anterior descending; RCA, right coronary artery

A provisional diagnosis of acute MI was formulated based on ongoing chest pain, dynamic EKG changes, rising troponin I on serial monitoring, and echocardiographic findings. The patient was started on a heparin drip and was medically managed with aspirin 325 mg orally, metoprolol 25 mg orally, and sublingual nitroglycerin 0.4 mg. The patient was transferred immediately to the cardiac catheterization lab for left heart catheterization with angiography. Coronary angiography showed 99% ostial left anterior descending (LAD) artery stenosis, mild luminal irregularities in the left circumflex artery, and right coronary artery (RCA) dominance. There was no stenosis noted in the left main artery and RCA (Figure 3).

**Figure 4:**
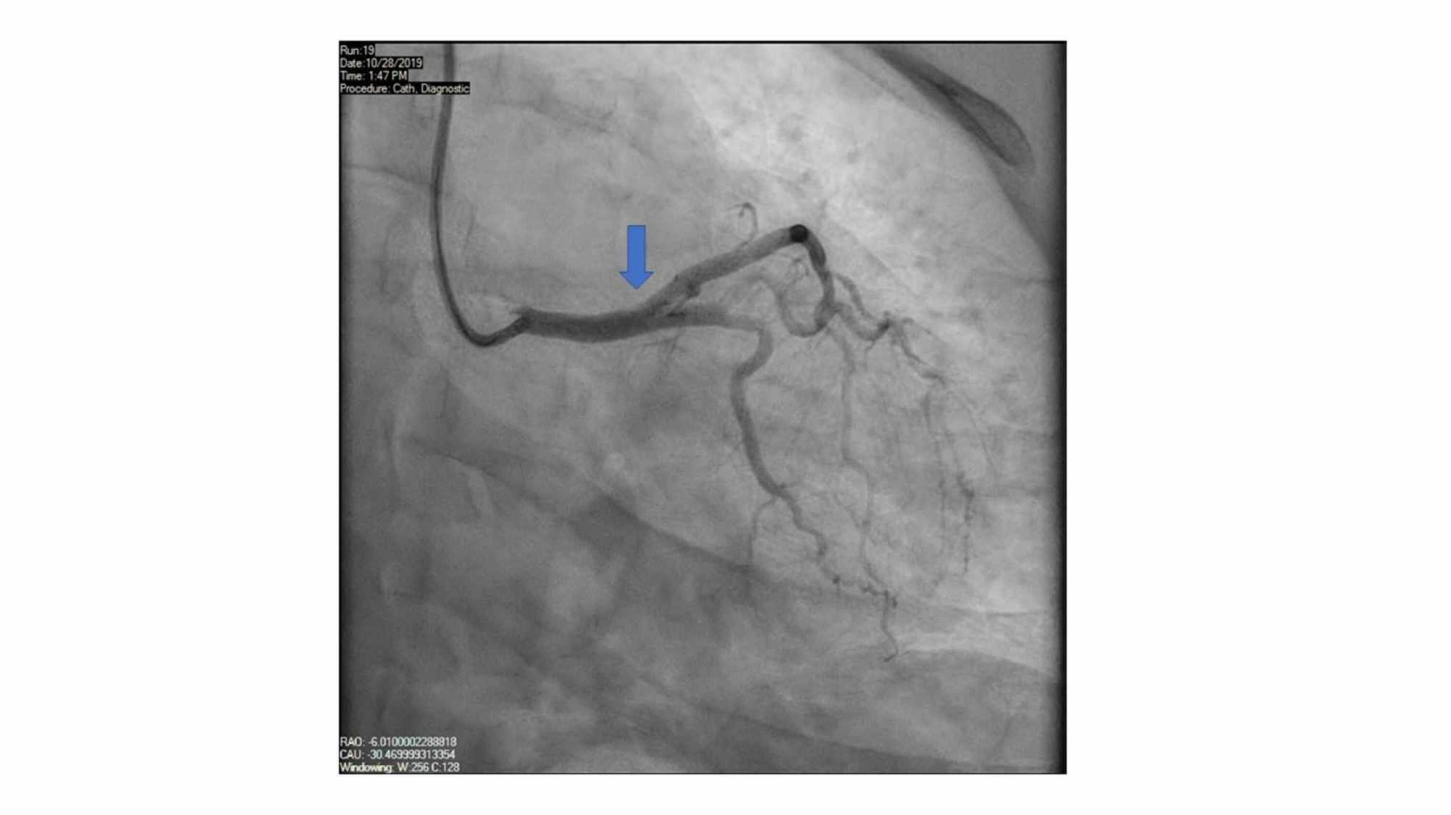
Post-intervention angiography. The blue arrow points to restoration of blood flow after drug eluting stent placement in the ostial LAD artery LAD, left anterior descending

A drug eluting stent (DES) was placed to the ostial LAD artery without any complications (Figure 4). 

She was discharged on oral medicines (aspirin 81 mg daily, atorvastatin 80 mg daily, metoprolol 50 mg BID, nitroglycerine 0.4 mg sublingual as needed, and prasugrel 10 mg daily) for secondary prevention. Dual antiplatelet therapy (aspirin and prasugrel) was continued for one year.

## Discussion

Polycystic ovarian syndrome is one of the most common endocrine problems in women of childbearing group with a prevalence ranging from 4% to 12% globally [1-3]. PCOS can be diagnosed based on four key diagnostic features (ovulatory dysfunction, hirsutism, hyperandrogenism, and multiple ovarian cysts on ultrasound) [1, 6]. It is the most frequent cause of anovulatory infertility, hirsutism, metabolic abnormalities, and also increases long-term risk for development of type 2 diabetes mellitus, endometrial carcinoma, obesity, anxiety, depression, and possibly cardiovascular disease [1, 3]. 

Myocardial infarction is the leading cause of death in women worldwide [3]. Postmenopausal women were affected the most, but it is also found to be increasing in younger women [7]. In younger women, causes of acute MI include arterial plaque, microvascular dysfunction, vasospasm, or spontaneous coronary artery dissection [3]. PCOS has an increased risk of coronary artery disease due to chronic inflammation, oxidative stress, impaired fibrinolysis, endothelial dysfunction, increased intimal thickness, increased coronary artery calcification, and impaired pulse wave velocity [8].

The association of PCOS with metabolic syndrome (including increased androgen, estrogen, homocysteine level, insulin) further increases the risk of CAD [2-4, 7, 9-10]. Both obese and nonobese patients with PCOS are found to be at increased risk [11]. Oral contraceptive pills further increase the risk of thromboembolic events and MI as it alters almost all the homeostatic parameters including plasma level of coagulation factors, anticoagulants proteins, and proteins involved in fibrinolytic pathways [10]. Due to the association with multiple risk factors, studies have found two-fold increased risk of CAD in patients with PCOS compared to women without PCOS [5]. 

As PCOS is associated with a range of reproductive, obstetric, metabolic and psychosocial features, management should focus on multiple aspects like metabolic, reproductive, and psychosocial features [3, 8]. The patient should also be monitored and managed for long-term metabolic complications [11-12]. 

## Conclusions

Polycystic ovarian syndrome is a common and increasingly challenging endocrine problem in women of childbearing age. Despite the association of PCOS with multiple comorbidities, this medical condition is not treated properly due to a lack of awareness and treatment guidelines. Patients with PCOS are found to be at increased risk for MI, stroke, and aortic dissection. The purpose of our case report is to raise awareness regarding the association of PCOS with MI, and our focus as a physician should provide emotional and psychosocial support to the patient. In conclusion, this case suggests an association of PCOS with MI. Further studies are needed to decrease the risk of cardiovascular disease in PCOS patients. Proper evidence-based guidelines are needed for appropriate prevention and management to reduce morbidity and mortality associated with it.
